# Specific pre-injury migraine characteristics associated with worse concussion outcomes

**DOI:** 10.1371/journal.pone.0345374

**Published:** 2026-03-26

**Authors:** Katelyn Tourigny, Faly Golshan, Carrie Esopenko, Jenna Francisco, Marla Mickleborough

**Affiliations:** 1 Department of Psychology and Health Studies, University of Saskatchewan, Saskatoon, Saskatchewan, Canada; 2 Department of Rehabilitation and Human Performance, Icahn School of Medicine at Mount Sinai, New York, United States of America; 3 College of Medicine, University of Saskatchewan, Saskatoon, Saskatchewan, Canada; University of Virginia, UNITED STATES OF AMERICA

## Abstract

**Objectives:**

We examined retrospectively recalled characteristics of pre-injury migraine and their association with retrospectively reported post-concussion outcomes.

**Methods:**

Data for this study was collected based on self-reported recall from a cross-sectional survey design, distributed online via the Prolific platform. Eligible participants (N = 271) completed the Migraine Disability Assessment Test, Rivermead Post-Concussion Symptoms Questionnaire, Quality of Life in Neurological Disorders short-form, and self-reported return to activities. A MANCOVA compared retrospectively reported concussion outcomes between individuals with and without a self-reported pre-injury migraine diagnosis. Partial correlations assessed associations between retrospectively recalled migraine characteristics and post-concussion outcomes.

**Results:**

Compared to those without a self-reported history of migraine (n = 208), individuals with a self-reported pre-injury migraine diagnosis (n = 61) retrospectively reported significantly greater severity of earlier (p = .007) and later (p = .032) cluster post-concussion symptoms, delayed return to work/school (p = .050) and sports (p < .001), and lower quality of life in the stigma (p < .001) subdomain. Retrospectively reported pre-injury migraine-related functional impairment was significantly correlated with recalled severity of earlier (r = .37) and later (r = .31) cluster post-concussion symptoms, return to sports (r = .29), and quality of life in the social role 1 subdomain (r = −.35). Retrospective reports of pre-injury migraine severity were significantly correlated with both earlier (r = .43) and later (r = .34) cluster post-concussion symptom severity, and lower quality of life in the anxiety (r = .45), depression (r = .46), dyscontrol (r = .38), fatigue (r = .32), wellbeing (r = −.32), sleep (r = .35), social role 1 (r = −.48), and cognition 2 (r = −.37) subdomains.

**Conclusions:**

Individuals with a retrospectively reported history of migraine recalled worse concussion outcomes than those without a migraine history. Additionally, more unfavourable recalled pre-injury migraine characteristics were associated with reports of more adverse post-concussion outcomes. These findings highlight the potential value of assessing individuals’ experience with pre-injury migraine when evaluating post-concussion challenges, and the need for future research to examine these relationships using longitudinal designs and objective clinical measures.

## Introduction

Determining the risk factors that increase vulnerablity to worse outcomes following a concussion remains an important public health concern [1]. Concussions occur when a hit to the head, neck, or body causes the brain to shift rapidly within the skull [2]. It is estimated that approximately 6 in every 1000 people worldwide experience a concussion annually, although this likely underestimates the true incidence [3]. Following a concussion, individuals can experience a range of somatic, cognitive, and emotional symptoms such as headaches, dizziness, forgetfulness, poor concentration, and depression [4]. Approximately 80–90% of individuals recover from post-concussion symptoms within 7–10 days [5]; however, 10–20% experience symptoms that extend beyond the typical recovery period (i.e., > 4 weeks) [6].

Migraine is the third most prevalent disorder globally [7], affecting approximately 11.8% of the population (13.8% females and 6.9% males) [8]. Additionally, migraine is the third highest cause of disability worldwide among individuals under 50 [9]. People with migraine report high levels of functional impairment and decreased quality of life (QoL) both during and between their headache attacks [10]. Interestingly, premorbid migraine is a pre-injury risk factor associated with prolonged recovery periods following a concussion [11–13]. A history of migraine has been associated with a greater likelihood of acute post-concussion symptoms in both the general adult population [14] and following sport-related injuries [15]. However, a recent systematic review reports mixed results regarding the relationship between pre-existing migraine and concussion outcomes [16]. Specifically, studies with larger samples and low bias did find that individuals with migraine experience greater post-concussion symptom severity [13], longer symptom duration [17–18], and delayed return to activities [19], while other studies found no significant associations [20–23]. The review concludes that a subgroup of athletes with pre-injury migraine may face higher risks of worse outcomes [16].

While prior research suggests a link between pre-injury migraine and persistent post-concussion symptoms, no studies have specifically examined whether particular characteristics of pre-injury migraine are associated with worse concussion outcomes. This study aimed to investigate associations between pre-injury migraine status and characteristics, and retrospectively reported post-concussion outcomes. We hypothesized that individuals with a self-reported history of pre-injury migraine would recall greater severity and longer duration of post-concussion symptoms, as well as lower QoL following the injury, compared to those without migraine. Additionally, we hypothesized that greater retrospectively recalled migraine severity, frequency, and functional impairment would be associated with worse post-concussion symptom severity, prolonged recovery, and lower QoL.

## Materials and methods

### Ethics

Prior to the start of this study involving human participants, ethical approval was obtained from the University of Saskatchewan’s Psychology Research Ethics Committee (Psy-REC #22−019). The study was conducted in accordance with the local legislation and institutional requirements. Participation was voluntary, and all participants were 18 years of age or older at the time of the study. Given the anonymous nature of the online survey and the absence of personally identifiable information, written or oral consent was not obtained. Instead, participants were presented with an online consent form outlining the nature of the study, their rights as participants, including the right to withdraw and how to do so, and data handling procedures. The form stated that clicking the ‘Next’ button to begin the survey and subsequently completing and submitting their responses implied free and informed consent and indicated that participants understood the outlined conditions of participation. Following submission of the survey, participants were presented with a debriefing form that reiterated the purpose of the study and included instructions on how to withdraw their data if they wished to do so.

### Study design

The current study employed an online, survey-based, retrospective cross-sectional design in which participants reported on their pre-injury migraine experience as well as outcomes and recovery patterns following their most recent concussion. All study materials were delivered in English. Participants were recruited via the Prolific platform, which predominately hosts English-proficient users. However, no formal screening for English fluency was conducted.

### Participants

The study utilized Prolific, an online research platform, to screen for eligible participants. Prolific advertised the study to participants who previously responded ‘yes’ to Prolific’s pre-screening question ‘have you ever had an injury to the head that caused you to be knocked out and/or dazed and confused for a period of time?’ completed when registering for the platform. After reading a description of the study on Prolific, participants were directed to a link to complete the online survey on SurveyMonkey. Study recruitment and data collection occurred between February 27^th^, 2023 and March 3^rd^, 2023. To be included in data analyses, participants either reported that they were diagnosed with a concussion by a healthcare professional following the head injury, or self-reported that they have had at least one concussion in their lifetime. Participants who indicated more severe injuries diagnosed by a healthcare professional (e.g., a hematoma, contusion, skull fracture, etc.) and/or self-reported having zero concussions in their lifetime were excluded from analyses.

A total of 305 individuals participated in the study from different countries (Table 1) and received compensation according to Prolific’s standard rates for completing the survey. Thirty-four participants were excluded due to either no lifetime concussion history (n = 13) or a diagnosis of a more severe head injury (n = 21), resulting in a final sample of 271 participants. Participants were categorized based on pre-injury migraine status: those with a self-reported diagnosis of migraine disorder by a healthcare professional prior to their concussion (n = 61; 42 men, 18 women, aged 19–58; M = 28.5, SD = 8.7), and as no-migraine individuals (n = 208; 133 men, 73 women, aged 18–78; M = 29.2, SD = 9.4) (Table 1).

**Table 1:** Sociodemographic characteristics of participants.

Sample characteristic	Total sample (N = 271)		Migraine (*n* = 61)		No-migraine (*n* = 208)	
	*n*	%	*n*	%	*n*	%
Sex						
Male	176	65.2	42	68.9	133	63.9
Female	91	33.7	18	29.5	73	35.1
Intersex	1	0.4	0	0	1	0.5
Prefer not to disclose	2	0.7	1	1.6	1	0.5
**Gender**						
Man	169	62.6	40	65.6	128	61.5
Woman	93	34.1	18	29.5	74	35.6
Other	6	2.2	1	1.6	5	2.4
Prefer not to disclose	1	0.4	1	1.6	0	0
Missing Data	2	0.7	1	1.6	1	0.5
**Ethnicity**						
White/European	200	74.1	42	68.9	157	75.5
Latin American	31	11.5	4	6.6	27	13.0
Black/African/Caribbean	29	10.7	15	24.6	14	6.7
Southeast Asian	5	1.9	0	0	5	2.4
South Asian	1	0.4	0	0	1	0.5
Missing Data	4	1.5	0	0	4	1.9
**Highest level of education**						
Some high school	7	2.6	2	3.3	5	2.4
Completed high school	37	13.7	9	14.8	28	13.5
Some college/university	67	24.8	12	19.7	54	26.0
Apprentice training and trades	9	3.3	3	4.9	6	2.9
Completed college/university	86	31.9	21	34.4	65	31.3
Some graduate education	14	5.2	3	4.9	11	5.3
Completed graduate education	33	12.2	6	9.8	27	13.0
Professional degrees	17	6.3	5	8.2	12	5.8
**Self-reported Concussion**	112		19		91	
**Physician Diagnosed Concussion**	159		42		117	
**Total number of concussions**						
1	130	48.1	24	39.3	105	50.5
2	79	29.3	19	31.1	60	28.8
3+	60	22.2	17	28.0	43	20.3
Missing data	1	0.4	1	1.6	1	0.4
**Cause of most recent concussion**						
Fall	137	50.7	30	49.2	107	51.4
Sports injury	72	26.7	19	31.1	53	25.4
Motor vehicle accident	31	11.5	7	11.5	23	11.1
Domestic violence	4	1.5	1	1.6	3	1.4
Blast or explosion	2	0.7	1	1.6	1	0.5
Other	20	7.4	2	3.3	18	8.7
Missing data	4	1.5	1	1.6	3	1.4
**Time since most recent concussion**						
Less than 3 months	20	7.4	3	4.9	17	8.2
3 months – 1 year	71	26.3	15	24.6	56	26.9
2–5 years	78	28.9	26	42.6	51	24.5
6–10 years	43	15.9	10	16.4	33	15.9
11–15 years	25	9.3	3	4.9	22	10.6
16–20 years	13	4.8	1	1.6	12	5.8
21 + years	14	5.2	1	1.6	13	6.3
Missing data	6	2.2	2	3.3	4	1.9
**History of repetitive head impacts**	42	15.6	13	21.3	29	13.9
Missing data	1	0.4	0	0	1	0.5
**Mental Health Condition**						
Anxiety	94	34.8	27	44.3	67	32.2
Depression	88	67.4	30	49.2	57	27.4
Attention Deficit						
Attention Deficit Disorder	32	11.9	10	16.4	22	10.6
Attention Deficit Hyperactivity Disorder	27	10.0	7	11.5	20	9.6
Learning Disability	7	2.6	2	3.3	5	2.4
**Family History of Migraine**						
Yes	98	36.3	31	50.8	67	32.2
Unsure	59	21.9	15	24.6	43	20.7

The participants were from different countries including Poland, Portugal, South Africa, Mexico, and the United Kingdom. Smaller percentages of participants reported currently residing in other countries around the world, including Italy, Spain, Greece, Hungary, England, Chile, Germany, Czech Republic, Estonia, Denmark, Scotland, United States of America, France, Australia, Finland, Norway, Latvia, Northern Ireland, Slovenia, Canada, Belgium, and Switzerland.

### Measures

#### The Migraine Disability Assessment Test (MIDAS).

The MIDAS assesses the degree to which migraine impedes a person’s ability to perform daily tasks and function normally, and was used to determine pre-injury migraine-related functional impairment, headache frequency, and severity. It is a widely used measure that has been psychometrically validated [24–26]. The 5-item MIDAS was modified so that participants were required to numerically report the number of days in the last three months *prior to their injury* that they were impaired (e.g., 5 days not doing household work due to headaches). The numbers were summed for all five questions and evaluated based on a scale: a score of 0–5 indicates ‘Little or No Disability,’ 6–10 is ‘Mild Disability,’ 11–20 is ‘Moderate Disability,’ while 21 + indicates ‘Severe Disability.’ Participants were required to respond to two questions to determine the frequency and severity of their pre-injury migraine (i.e., on how many days in the 3 months *prior to your injury* did you have a headache, and on a scale of 0–10, on average how painful were these headaches?).

#### The Rivermead Postconcussion Symptoms Questionnaire (RPQ).

The RPQ and an added question were used to assess the severity and duration of post-concussion symptoms. The RPQ is a 16-item self-reported questionnaire [27] that measures the presence and severity of commonly experienced somatic, cognitive, and emotional symptoms post-concussion [4]. Participants rate each symptom on a likert scale, indicating the degree to which it has been more of a problem after the injury compared to before, from ‘not experienced’ (0) to ‘severe problem’ (4). These ratings are summed in two groups, the RPQ-3 and RPQ-13, based on a modified, validated scoring system [28]. The first 3 items on the RPQ (headaches, feelings of dizziness, and nausea) make up the first group (RPQ-3) and represent an earlier cluster of post-concussion symptoms, whereas the remaining 13 items (such as noise sensitivity, sleep disturbance, fatigue, etc.) make up the second group (RPQ-13) and represent a later cluster of post concussion symptoms.

In the current study, the RPQ was modified such that participants were asked to retrospectively recall and rate symptoms experienced following their most recent concussion, rather than in the past 24 hours, as per the standard RPQ format. This modification was necessary to accommodate the retrospective design of the study. The RPQ is a widely used measure of post-concussion symptoms, with excellent internal consistency (*α* = .94) [29] and test-retest reliability (0.89 for RPQ-13 and 0.72 for RPQ-3, both p < .01) [28]. Although modifying the temporal frame may affect the precision of symptom severity estimates and limit direct comparisons with normative values or studies using the standard timeframe, the symptom domains assessed were unchanged. Accordingly, RPQ scores were interpreted as reflecting relative differences in retrospectively reported symptom severity between groups rather than precise estimates of symptom severity at a specific time point. In addition to rating the severity of each symptom on the RPQ, participants were asked to categorically report how long they experienced each symptom following their injury.

#### Return to previous activities.

Participants were asked to self-report, from the date of their most recent concussion, the number of days before they were fully cleared to return to work/school and sports. It is important to note that responses to these questions were based on participant estimates rather than documented clinical timelines. Some individuals reported that they never received formal medical clearance from a healthcare professional before returning to work/school or sports. Additionally, we recognize that without formal documentation, participants’ recall of events that occurred many years ago may be subject to recall bias.

#### Quality of Life in Neurological Disorders (Neuro-QoL) v1.0 Short Form.

The Neuro-QoL short form version 1.0 is a self-report measure that evaluates the generic health-related quality of life (HRQoL) symptoms, concerns, and issues in adults with neurological conditions, including brain injury, across 13 subdomains [30]. Participants responded to each item on a likert scale representing the intensity, frequency, and duration of each of the 13 HRQoL subdomains. For positively worded concepts (e.g., communication), a higher score is indicative of better (desirable) self-reported health. For the negatively worded concepts (e.g., anxiety), a higher score is indicative of worse (undesirable) self-reported health. Missing scores on the Neuro-QoL were approximated according to guidelines outlined in the user manual [31]. The measure has shown high levels of reliability and internal consistency [30].

### Statistical analyses

All data analyses were completed using SPSS 28.0. For Objective 1, a multivariate analysis of covariance (MANCOVA) was conducted to examine the relationship between pre-injury migraine status and retrospectively reported concussion outcomes. The independent variable was pre-injury migraine status (i.e., presence or absence of migraine disorder) and the dependent variables were concussion outcomes (i.e., recalled symptom severity, return to previous activities, and QoL following the injury). Biological sex and a self-reported history of anxiety and depression were included as covariates in the analysis based on prior research that suggests sex and mental health are associated with concussion recovery trajectories and migraine burden [32,33]. Chi-square analyses were conducted to compare the distribution of symptom duration categories (<1 week, 1–4 weeks, 1–3 months, > 3 months, or ongoing) between individuals with and without a self-reported pre-injury migraine diagnosis. To further examine the effect of sex within the migraine group, independent samples t-tests were conducted comparing males and females on key variables.

For Objective 2, associations between retrospectively reported pre-injury migraine characteristics (i.e., migraine-related functional impairment, frequency, and severity) and concussion outcomes (i.e., recalled symptom severity, return to previous activities, and QoL following the injury) were examined using partial correlations, controlling for biological sex and a self-reported history of anxiety and depression. An alpha level of p < .05 was interpreted as being significant.

## Results

### Objective 1: Differences in concussion outcomes dependent on migraine status

The MANCOVA revealed a significant main effect of pre-injury migraine status on concussion outcomes, Wilks’ Λ = .815, F_(17, 241)_ = 3.21, p < .001, partial η^2^ = .185, indicating significant differences in retrospectively reported concussion outcomes between individuals with and without migraine while controlling for biological sex, anxiety, and depression. As shown in Table 2, univariate effects and estimated marginal means revealed that participants with a self-reported pre-injury migraine diagnosis reported significantly greater severity of post-concussion symptoms as measured by RPQ scores, took significantly longer to return to work/school (M = 12.1 days) and sports (M = 23.2 days), and reported significantly lower QoL in the stigma subdomain compared to those without a history of pre-injury migraine. There were no significant group differences in the duration of post-concussion symptoms for either earlier, *X*^2^ (5, *N* = 268) = 7.967, *p* = .158, or later symptom clusters, *X*^2^ (5, *N* = 268) = 6.531, *p* = .258.

**Table 2:** Univariate effects and adjusted means for concussion outcomes in migraine and no-migraine groups, controlling for biological sex, anxiety, and depression.

	Estimated Marginal Means			Univariate Results		
Concussion Outcomes	Migraine *(n* = 57)	No-migraine *(n* = 205)				
Post-concussion Symptom Severity	M (SE)	M (SE)	95% CI [Lower-Upper]	F _(df1, df2)_	*p*	Partial η^2^
RPQ-3	9.1 (0.35)	8.0 (0.18)	[8.43-9.82], [7.67-8.39]	7.49 _(1, 257)_	.**007**	.028
RPQ-13	33.0 (1.40)	29.5 (0.73)	[30.21-35.73], [28.10-30.97]	4.63 _(1, 257)_	.**032**	.018
**Return to Previous Activities**	**M days (SE)**	**M days (SE)**	**95% CI** **[Lower-Upper]**	**F**_**(df1, df2)**_	***p***	**Partial η** ^**2**^
Work/School	25.8 (5.40)	13.7 (2.81)	[15.15-36.41], [8.16-19.22]	3.88 _(1, 257)_	**.050**	.015
Sports	46.5 (6.03)	23.3 (3.13)	[34.65-58.38], [17.15-29.39]	11.48 _(1, 257)_	<.**001**	.043
**Quality of Life**	**M (SE)**	**M (SE)**	**95% CI** **[Lower-Upper]**	**F**_**(df1, df2)**_	***p***	**Partial η** ^**2**^
Communication	20.8 (0.48)	21.6 (0.25)	[19.86-21.74], [21.07-22.05]	1.99 _(1, 257)_	.159	.008
Participation	22.8 (0.77)	22.7 (0.40)	[21.25-24.28], 21.90-23.47]	0.01 _(1, 257)_	.930	.000
Anxiety	20.7 (0.90)	21.9 (0.47)	[18.96-22.51], [20.95-22.79]	1.23 _(1, 257)_	.268	.005
Depression	19.7 (1.10)	19.0 (0.57)	[17.51-21.85], [17.87-20.12]	0.30 _(1,257)_	.586	.001
Dyscontrol	17.7 (0.89)	18.6 (0.46)	[15.96-19.45], [17.71-19.53]	0.83 _(1, 257)_	.364	.003
Fatigue	24.3 (0.99)	22.5 (0.51)	[22.39-26.29], [21.51-23.53]	2.63 _(1, 257)_	0.106	.010
Wellbeing	27.7 (1.11)	27.7 (0.58)	[25.47-29.84], [26.60-28.87]	0.00 _(1, 257)_	.950	.000
Sleep	21.5 (0.83)	20.2 (0.43)	[19.90-23.17], [19.32-21.02]	2.09 _(1, 257)_	.149	.008
Stigma	14.2 (0.73)	11.1 (0.38)	[12.71-15.59], [10.36-11.86]	13.41 _(1, 257)_	**<.001**	.050
Social Roles 1	15.2 (0.57)	16.2 (0.30)	[14.07-15.64], [15,64-16.80]	2.58 _(1, 257)_	.110	.010
Social Roles 2	11.8 (0.53)	11.9 (0.27)	[10.73-12.81], [11.35-12.43]	0.04 _(1, 257)_	.847	.000
Cognition 1	12.8 (0.55)	13.2 (0.29)	[11.67-13.84], [12.64-13.77]	0.52 _(1, 257)_	.471	.002
Cognition 2	15.6 (0.47)	15.4 (0.24)	[14.67-16.50], [15.05-16.00]	0.01 _(1, 257)_	.909	.000

M = Estimates Marginal Mean; SE = Standard Error; CI = Confidence Intervals. All means are adjusted for biological sex, anxiety, and depression. The RPQ-3 represents earlier cluster post-concussion symptom severity, whereas the RPQ-13 provides a measure of later cluster post-concussion symptom severity. Significant p-values in bold font.

**Fig 1:**
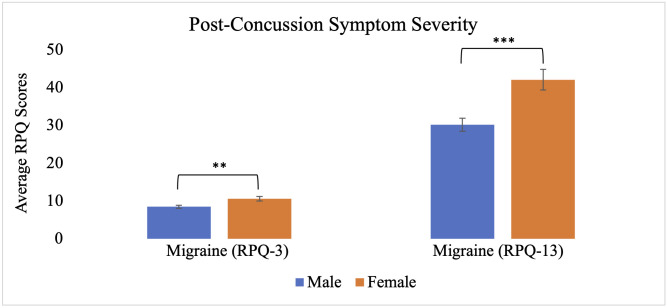
Post-concussion symptom severity for individuals with migraine based on biological sex. Each bar represents the average score on the Rivermead Post-Concussion Symptoms Questionnaire for males and females with migraine. Error bars represent standard error of the mean. The RPQ-3 represents earlier cluster post-concussion symptom severity, whereas the RPQ-13 provides a measure of later cluster post-concussion symptom severity. ** p < .01 (two-tailed), *** p < .001 (two-tailed).

**Fig 2:**
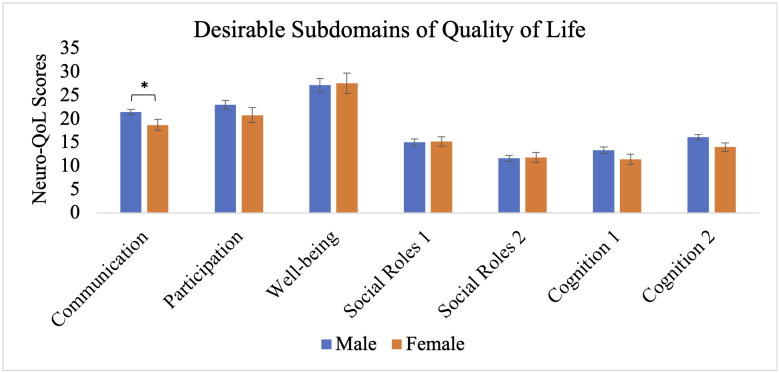
Desirable subdomains of quality of life for individuals with migraine based on biological sex. Error bars represent standard error of the mean. * p < .05 (two-tailed).

**Fig 3:**
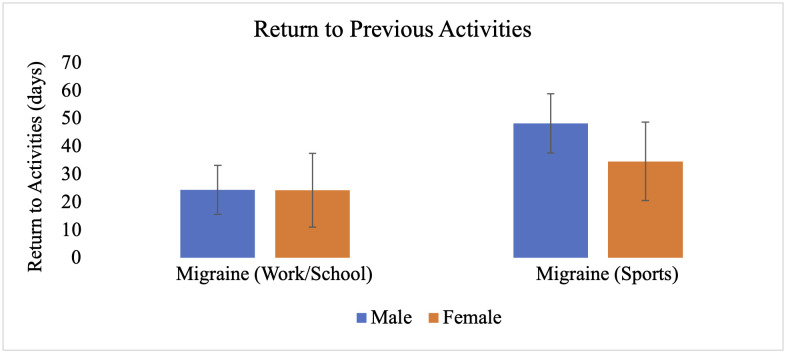
Return to previous activities for individuals with migraine based on biological sex. Each bar represents the average number of days that it took individuals to return to work/school and sports following their concussion. Error bars represent standard error of the mean.

**Fig 4:**
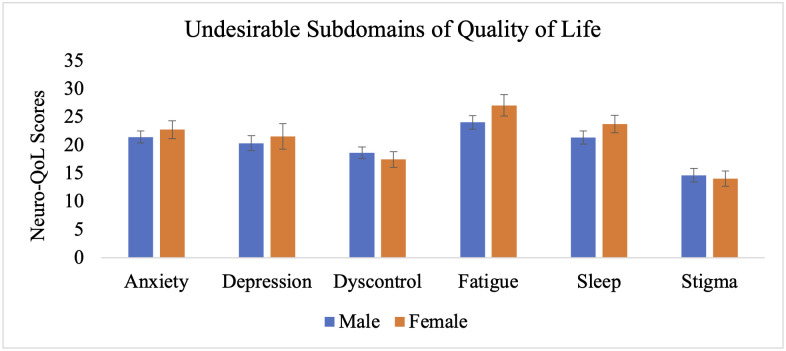
Undesirable subdomains of quality of life for individuals with migraine based on biological sex. Error bars represent standard error of the mean.

The MANCOVA also revealed significant main effects of biological sex, Wilks’ Λ = .848, F_(17, 241)_ = 2.53, p < .001, partial η^2^ = .152, and depression, Wilks’ Λ = .881, F_(17, 241)_ = 1.909, p = .018, partial η^2^ = .119. Independent samples t-tests within the migraine group revealed that females reported significantly greater severity of earlier cluster post-concussion symptoms (M = 10.6, SE = .59) compared to males (M = 8.5, SE = .39), t(58) = −2.93, p < .01, as well as later cluster symptoms (females: M = 42.1, SE = 2.8; males: M = 30.2, SE = 1.7), t(58) = −3.75, p < .001 (Fig 1). Females also reported significantly lower health-related quality of life in the communication subdomain (M = 18.7, SE = 1.2) relative to males (M = 21.5, SE = .54), t(58) = 2.45, p < .05 (Fig 2). No significant sex differences were observed in time to return to work/school (females: M = 24.3, SE = 13.2; males: M = 24.4, SE = 8.8), t(58) = 0.01, p = .994, return to sports (females: M = 34.6, SE = 14.1; males: M = 48.2, SE = 10.6), t(58) = 0.73, p = .471 (Fig 3), or other subdomains of quality of life (Fig 4).

### Objective 2: Associations between pre-injury migraine characteristics and concussion outcomes

As shown in Table 3, partial correlations controlling for biological sex, anxiety, and depression revealed that greater retrospectively reported pre-injury migraine-related functional impairment was associated with the recall of more severe earlier (r_(48)_ =.37, p < .01) and later (r_(48)_ =.31, p < .05) cluster post-concussion symptoms, longer time to return to sports (r_(48)_ =.29, p < .05), and lower QoL in the social role 1 subdomain (r_(48)_ = −.35, p < .05). Retrospective ratings of pre-injury migraine severity were positively correlated with both earlier (r_(48)_ =.43, p < .01) and later (r_(48)_ =.34, p < .05) cluster post-concussion symptom severity and negatively correlated with QoL in the wellbeing (r_(48)_ = −.32, p < .05), social role 1 (r_(48)_ = −.48, p < .01), and cognition 2 (r_(48)_ = −.36, p < .01) subdomains. Pre-injury migraine severity was also positively correlated with QoL in the anxiety (r_(48)_ =.45, p < .01), depression (r_(48)_ =.46, p < .01), dyscontrol (r_(48)_ =.38, p < .01), fatigue (r_(48)_ =.32, p < .05), and sleep (r_(48)_ =.35, p < .05) subdomains.

**Table 3:** Partial correlations between pre-injury migraine characteristics and concussion outcomes, controlling for biological sex, anxiety, and depression.

	Pre-Injury Migraine MIDAS Scores		
	Functional impairment (*n* = 53)	Migraine frequency (*n* = 53)	Migraine severity (*n* = 53)
**RPQ**			
RPQ-3	.37**	.18	.43**
RPQ-13	.31*	−.01	.34*
**Return to Activities**			
Work/School	.12	−.10	.17
Sports	.29*	.04	.18
**Neuro-QoL**			
Communication	−.13	.08	−.21
Participation	−.09	−.11	−.27
Anxiety	.24	.16	.45**
Depression	.12	.04	.46**
Dyscontrol	.13	−.01	.38**
Fatigue	.09	.09	.32*
Wellbeing	−.15	−.20	−.32*
Sleep	.16	.12	.35*
Stigma	.22	−.13	.26
Social Roles1	−.35*	.11	−.48**
Social Roles2	−.16	−.19	−.24
Cognition1	−.03	−.00	−.25
Cognition2	−.03	−.02	−.37**

The RPQ-3 represents earlier cluster post-concussion symptom severity, whereas the RPQ-13 provides a measure of later cluster post-concussion symptom severity. Significant correlations in bold, * p < .05 (two-tailed), ** p < .01 (two-tailed).

## Discussion

The present study used a retrospective, cross-sectional online survey to examine how recalled pre-injury migraine characteristics, including headache severity, frequency, and migraine-related functional impairment, were associated with recalled post-concussion symptom severity, symptom duration, return to activities, and QoL. The retrospective design limits causal interpretations of the relationship between pre-injury migraine characteristics and concussion outcomes. However, the use of consistent methods across all participants may help reduce some limitations associated with between-group comparisons, as all groups are assumed to have similar levels of recall bias.

In line with previous research, individuals with a history of migraine recalled greater post-concussion symptom severity, delayed return to work/school and sports, and lower QoL in the stigma subdomain compared to those without a migraine history. Our findings extend prior research by suggesting that retrospectively recalled pre-injury migraine severity and functional impairment were associated with more severe post-concussion symptoms, delayed return to sports, and greater reductions in QoL across a number of subdomains. The following sections explore potential mechanisms underlying this relationship, implications for QoL, and the importance of considering sex differences in migraine and concussion research.

### Possible mechanisms underlying the relationship between migraine and concussion

Consistent with previous research [34–37], our findings indicate that individuals with a pre-injury migraine diagnosis report greater post-concussion symptom severity, delayed return to previous activities, and lower QoL. While the mechanisms underlying this association remain complex, cortical spreading depression (CSD) may play a contributing role. CSD is a wave of neuronal depolarization across the cortex, followed by a period of neuronal suppression [38]. It is implicated in the pathophysiology of migraine aura and is thought to trigger the pain, nausea, and sensory sensitivities during migraine headaches [39].

Recent research suggests CSD may also influence concussion recovery. Animal studies show that CSD can occur after head injuries, altering cerebral blood flow, metabolism, and neural activity [40]. The changes in the brain caused by CSD may exacerbate and prolong post-concussion symptoms [41]. Although the relationship between CSD and concussion outcomes remains unclear, individuals with pre-existing migraine, who already experience CSD, may be more vulnerable to worse post-concussion outcomes compared to those without migraine. Future research should explore the role of CSD in this relationship and whether targeting CSD-related mechanisms could improve recovery in individuals with comorbid migraine and concussion.

### The impact of migraine and concussion on quality of life

Several factors may contribute to why individuals with migraine experience lower QoL following a concussion. For instance, the co-occurance of migraine and post-concussion symptoms may intensify overall symptom severity, as individuals in our study recalled. Increased symptom severity following a head injury can negatively impact mental health, interfere with daily functioning (e.g., completing household chores), and delay return to work, school, sports, or social activities – domains already known to be compromised and associated with reduced QoL in individuals with migraine [10,42–44]. Additionally, prior studies have shown that lower health-related QoL following a concussion is associated with increased post-concussion symptom severity [45] and duration [46].

These overlapping challenges may create a feedback loop, where reduced QoL contributes to prolonged or intensified symptoms for individuals managing both migraine and concussion. Although the relationship between pre-injury migraine and QoL following a concussion is multidimensional, our findings underscore the importance of effectively managing both migraine and post-concussion symptoms in clinical care to improve QoL and facilitate more efficient recoveries in this population.

### Considering sex differences in migraine and concussion research

Sex and gender are important factors to consider when examining the relationship between pre-injury migraine and concussion outcomes. Approximately 22% of our sample reported a diagnosis of pre-injury migraine, with roughly 30% being female. While both male and female participants with migraine reported significantly greater post-concussion symptom severity than those without migraine, consistent with previous literature [32], females with migraine in our study recalled significantly more severe concussion symptoms than males with migraine.

Although the mechanisms underlying these sex differences remain unclear, a systematic review suggests that hormonal differences in females at the time of injury and during recovery could play a role [47]. For example, women who sustain a mTBI during the luteal phase of their menstrual cycle, when progesterone levels are elevated, report more severe post-concussion symptoms and lower quality of life during recovery compared to women using oral contraceptives and those who are injured during the follicular phase of their cycle [48].

Additionally, it is important to note that hormonal cycles are known to influence the frequency, severity, and debilitating nature of migraine headaches, particularly around menstruation [33]. These overlapping hormonal effects may contribute to both increased migraine burden and more complex post-concussion recovery in females. Together, these findings highlight the importance of considering biological sex when evaluating concussion outcomes in individuals with migraine. A better understanding of sex-specific mechanisms may help inform tailored clinical management strategies to support recovery in this population.

### Limitations

Our study has several limitations that should be considered when interpreting the findings. Most notably, the retrospective, online, single-point contact design relied on self-reported data, which may introduce recall bias, particularly regarding pre-injury migraine characteristics and post-concussion outcomes. This concern is especially relevant for participants with injuries that occurred many years ago, as the accuracy of symptoms and recovery recall tends to diminish over time. While this design limits the ability to draw causal inferences about the relationship, the use of consistent methodology across all participants strengthens between-group comparability as all groups of participants are assumed to have similar levels of recall bias and offers preliminary insights that merit further investigation. Not all participants sought follow-up care post-injury and were not formally cleared to return to work/school or sports. This highlights the need for improved education and access to post-concussion care, especially for individuals with premorbid conditions like migraine, who may be at increased risk of prolonged or more severe recovery challenges. Certain variables, such as the number of days to full recovery, were especially prone to recall difficulty, and the self-report format led to some incomplete responses. Additionally, the symptom duration categories used in the survey (e.g., 1–4 weeks, 1–3 months) may have caused confusion, as some participants may have interpreted these categories as equivalent, possibly leading to minor misclassification of symptom duration. Another limitation is the absence of formal screening for English language fluency. Although the study was delivered in English and Prolific states that most users on the Prolific platform are proficient in English, we cannot rule out the possibility that limited fluency may have influenced participants’ understanding or interpretation of the survey items. Furthermore, we did not control for total number of concussions or a history of repetitive head impacts, which have been shown to influence concussion recovery patterns [49].

These limitations provide important directions for future studies, particularly studies that involve real-time data collection and collaboration with healthcare professionals to obtain more objective and detailed assessments of post-concussion symptom severity, duration, and QoL. Despite these limitations, our findings have important clinical implications and emphasize the need for future studies to further explore the relationship between pre-injury migraine, its characteristics, and concussion outcomes. Further work should examine how specific migraine characteristics influence concussion outcomes, with the goal of informing targeted interventions to better support individuals with migraine following a concussion.

## Conclusion

Individuals with pre-injury migraine retrospectively report greater post-concussion symptom severity, delayed return to work/school and sports, and lower quality of life related to stigma following a concussion than those without a history of migraine disorder. Additionally, our study provides some of the first evidence linking specific characteristics of pre-injury migraine to worse concussion outcomes. Specifically, the retrospective recall of more severe headaches and greater migraine-related functional impairment prior to a concussion was associated with the recall of more severe post-concussion symptoms, delayed return to sports, and reduced quality of life following the injury. Our findings highlight the need for future research to consider specific characteristics of a pre-existing migraine diagnosis when evaluating and managing individuals who sustain concussions. A more comprehensive pre-injury migraine assessment following a head injury may also enhance clinical management, potentially leading to improved outcomes for concussion patients. The dataset for this study can be found in the S1 Data.

## Supporting information

S1 Data Dataset. (XLSX)
